# Development of Computational Tools for the Inference of Protein Interaction Specificity Rules and Functional Annotation Using Structural Information

**DOI:** 10.1002/cfg.304

**Published:** 2003-07

**Authors:** Fabrizio Ferrè, Allegra Via, Gabriele Ausiello, Barbara Brannetti, Andreas Zanzoni, Manuela Helmer-Citterich

**Affiliations:** Centre for Molecular Bioinformatics Department of Biology University of Rome Tor Vergata, Via Della Ricerca Scientifica Rome 00133 Italy

## Abstract

Relatively few protein structures are known, compared to the enormous amount of sequence data produced in the sequencing of different genomes, and relatively few protein complexes are deposited in the PDB with respect to the great amount of interaction data coming from high-throughput experiments (two-hybrid or affinity purification of protein complexes and mass spectrometry). Nevertheless, we can rely on computational techniques for the extraction of high-quality and information-rich data from the known structures and for their spreading in the protein sequence space. We describe here the ongoing research projects in our group: we analyse the protein complexes stored in the PDB and, for each complex involving one domain belonging to a family of interaction domains for which some interaction data are available, we can calculate its probability of interaction with any protein sequence. We analyse the structures of proteins encoding a function specified in a PROSITE pattern, which exhibits relatively low selectivity and specificity, and build *extended* patterns. To this aim, we consider residues that are well-conserved in the structure, even if their conservation cannot easily be recognized in the sequence alignment of the proteins holding the function. We also analyse protein surface regions and, through the annotation of the solvent-exposed residues, we annotate protein surface patches via a structural comparison performed with stringent parameters and independently of the residue order in the sequence. Local surface comparison may also help in identifying new sequence patterns, which could not be highlighted with other sequence-based methods.


**Comparative and Functional Genomics**
*Comp Funct Genom* 2003; **4**: 416–419.
Published online 18 July 2003 in Wiley InterScience (www.interscience.wiley.com). **DOI:** 10.1002/cfg.304

**Conference Review**

### Development of computational tools for the inference of protein interaction specificity rules and functional annotation using structural information

Fabrizio Ferrè[#], Allegra Via[#], Gabriele Ausiello, Barbara Brannetti, Andreas Zanzoni and Manuela Helmer-Citterich*
*Centre for Molecular Bioinformatics, Department of Biology, University of Rome, Tor Vergata, Rome, Italy*

**Correspondence to: Manuela Helmer-Citterich, Centre for Molecular Bioinformatics, Department of Biology, University of Rome, Tor Vergata, Via Della Ricerca Scientifica, 00133 Rome, Italy. E-mail: citterich@uniroma2.it*

*[#] These authors contributed equally to this work.*

Received: 7 May 2003
Revised: 30 May 2003
Accepted: 30 May 2003

### Abstract

**Relatively few protein structures are known, compared to the enormous amount of sequence data produced in the sequencing of different genomes, and relatively few protein complexes are deposited in the PDB with respect to the great amount of interaction data coming from high-throughput experiments (two-hybrid or affinity purification of protein complexes and mass spectrometry). Nevertheless, we can rely on computational techniques for the extraction of high-quality and information-rich data from the known structures and for their spreading in the protein sequence space. We describe here the ongoing research projects in our group: we analyse the protein complexes stored in the PDB and, for each complex involving one domain belonging to a family of interaction domains for which some interaction data are available, we can calculate its probability of interaction with any protein sequence. We analyse the structures of proteins encoding a function specified in a PROSITE pattern, which exhibits relatively low selectivity and specificity, and build *extended* patterns. To this aim, we consider residues that are well-conserved in the structure, even if their conservation cannot easily be recognized in the sequence alignment of the proteins holding the function. We also analyse protein surface regions and, through the annotation of the solvent-exposed residues, we annotate protein surface patches via a structural comparison performed with stringent parameters and independently of the residue order in the sequence. Local surface comparison may also help in identifying new sequence patterns, which could not be highlighted with other sequence-based methods.** **Copyright © 2003 John Wiley & Sons, Ltd.**

**Keywords:** **bioinformatics; protein interaction; protein surface**

### The spot method to infer protein interaction specificity: structural information used to extract sequence rules

We select those protein–protein complexes involving one protein interaction domain (e.g. SH2, SH3, PDZ, WW domains) in the PDB, and analyse each family independently of the other families. Not all of the protein interaction domains can be successfully used with our method: it is important that the partner of the interaction is known to assume a conserved 3D structure (such as a polyproline tract or a C-terminus extended filament) and that enough interaction data is available. Interaction data can be provided in the form of sequences of interacting peptides. We rely on peptide sequences identified as binding partners in phage display and *peptide array* experiments [3,7].

We first identify, for each family of protein interaction modules, the residues involved in the

Copyright © 2003 John Wiley & Sons, Ltd.

binding. We then build a matrix whose columns represent the contacting residues in the interaction module and whose rows represent the contacting residues in the partner of the interaction (Figure 1). Interaction data about stable complexes of known sequence involving the interaction module can provide information to fill the elements of the table, representing the contact positions with the frequencies of the residues in the binding proteins or peptides. Such a matrix of frequencies of residue–residue contact pairs in established contact positions in the binding surface of stable experimental complexes can be used to infer the probability of interaction between a member of the interaction module family and a protein, even if no data are available about its specificity.

The SPOT (sequence prediction of target) procedure is now available on the web as iSPOT (*internet*SPOT) at: **http://cbm.bio.uniroma2.it/ispot/** for members of the SH3, PDZ and WW domain families [1].

### Analysis of protein structures to build *extended* PROSITE-like patterns with increased selectivity and specificity

Functional properties of a protein or a set of proteins can often be described as sequence patterns or motifs. Many sequence patterns match all and only the known true positive sequences, i.e. sequence of proteins sharing the function associated to the functional pattern. Given an uncharacterized protein

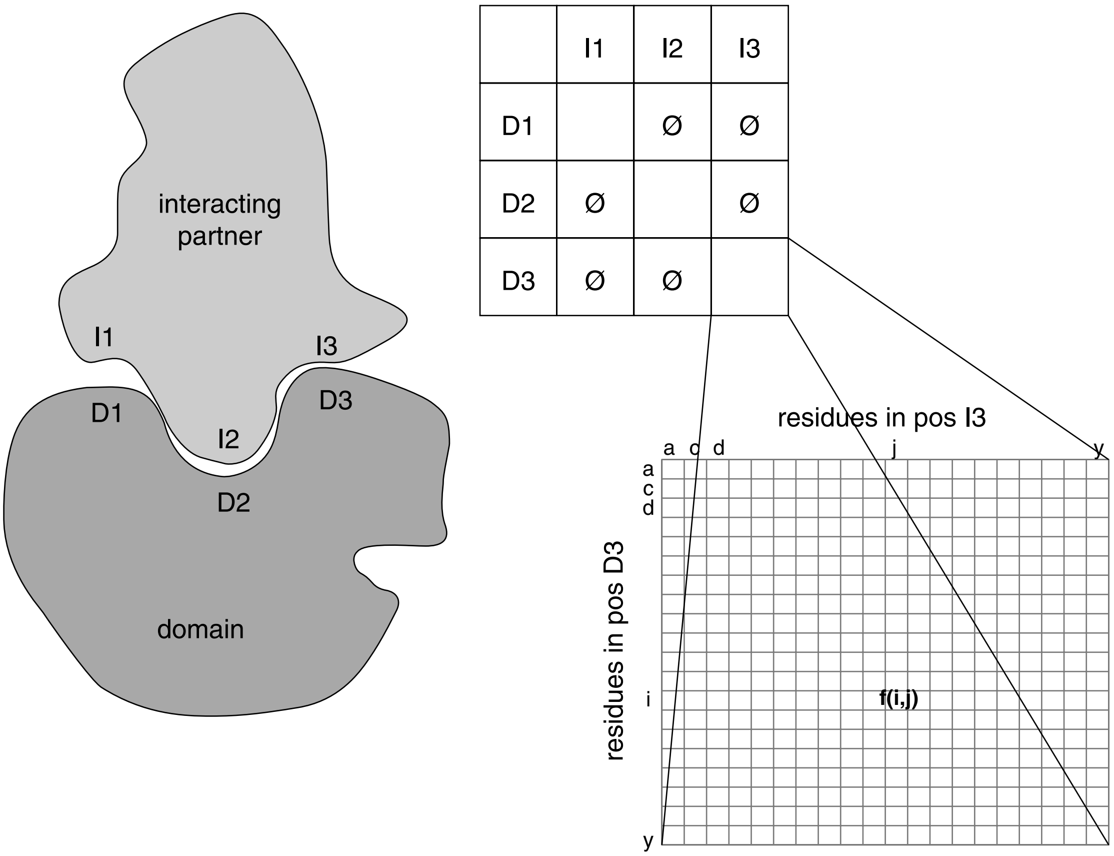

**Figure 1.** A simplified representation of an interaction involving two proteins. One domain (D) and one of its interactors (I) contact each other using only three contacting residues (D1, D2 and D3 on the domain, and I1, I2 and I3 on the interactor). We describe the interaction in the matrix drawn on the right, where the columns and the rows represent the contacting positions in the domain and in the interactor, respectively. In each contacting position (D1–I1, D2–I2 and D3–I3) we report the frequencies of the residues identified in stable domain–interactor complexes. Such frequencies can be used to evaluate the probability of interaction between the two proteins

Copyright © 2003 John Wiley & Sons, Ltd. 

whose function(s) is/are unknown, such patterns can be used for function inference with a high degree of reliability. However, a great number of patterns select false positives and/or miss known true positives. In this work, we explore the possibility of improving the discriminating power of poorly performing patterns, in order to obtain more reliable tools for sequence analysis and function inference.

Sequence patterns are usually obtained using conserved residues identified in a multiple sequence alignment of related proteins. It is possible that — at least in some cases — the weak specificity and/or sensitivity of a pattern is due to the lack of functional key residues in the sequence pattern. Indeed, while localized in space, functional residues may be dispersed along the protein sequences and therefore not easily detectable by means of multiple sequence alignment methods. In the present work, we focused on functional patterns displaying a weak predictive power and used protein structures to identify amino acids likely to be important for the function of such proteins. For each pattern that is considered, the true positive matching sequences for which the structure is known are collected. Such structures are then used to identify residues potentially directly involved in the protein function or indirectly relevant to that function. The sequence positions of such residues are used to build new sequence patterns. For the majority of the cases analysed it is possible to obtain new patterns displaying an improved ability to discriminate between true and false positives with respect to the original sequence patterns. The method allows the identification of amino acids that do not occupy conserved positions in a multiple sequence alignment. The addition of structural constraints to poorly performing patterns proved to be an effective way to enhance their predictive power. The new patterns can be used to scan protein sequence databases in the context of proteomics, in order to contribute to function assignment of newly sequenced proteins (Via *et al.*, manuscript in preparation).

### Analysis of protein surface: a database and a method for functional annotation

Deciphering protein function(s) will be one of the major tasks in the next years, due to the growing number of uncharacterized protein structures that will be available from several structural genomics projects [2]. Moreover, we believe that functional annotation may still be useful for already characterized proteins, in cases where more functions are encoded and not all of them have been analysed by biochemical or genetic experiments.

We built a procedure to infer functions of a protein, given its structure, by means of local structural similarities with characterized proteins. Since functions are often encoded by a small subset of residues that are close in space, and have access to the surface but are somehow protected from the environment (corresponding to surface clefts; [6]), we tried to automatically identify potential functional surface regions. We collected a set of functional sites from a non-redundant list of proteins of known structure from the PDB [8], and we mapped functional residues over these functional regions. Functional sites have been identified using a surface-scanning algorithm (SURFNET; [5]) that is able to automatically identify the largest surface clefts. Automatic procedures have been applied to map functional residues on these functional sites by means of the interaction with ligands co-crystallized with the protein, or by means of the match to the sequences of known functional motifs (using the PROSITE database; [4]). This database of functional sites can be used to infer the function(s) of a target protein, by looking for local structural similarities involving solvent-exposed residues.

An efficient structural comparison algorithm (Ausiello *et al.*, manuscript in preparation) allows the fast scanning of a structure against the collection of functional sites. The reliability of the overall procedure has been tested using several benchmark cases in which proteins with different sequence and/or fold share a similar functional site (results not shown). Moreover, since an all-vs.-all comparison was feasible due to the computational efficiency of the algorithm, we were able to compare a large dataset of putative protein functional sites. The results of this large-scale analysis can be retrieved through a web interface that accesses a relational database (Ferrè *et al.*, manuscript in preparation). Given the sequence- and/or topology-independent nature of the procedure, non-obvious local similarities can be detected, offering a tool that can be used to integrate, validate or negate

Copyright © 2003 John Wiley & Sons, Ltd. 

functional prediction results obtained by classical sequence alignment methods.

It has been found that in several cases, a global similarity between protein sequences cannot always be related to a functional similarity, since sometimes the residues of the active site are not conserved in the protein of unknown function. A possible application of our method for functional annotation can therefore be extended to reliable 3D models of proteins, when the sequence is conserved, but the function remains uncertain.

### Conclusions

Rules that can be applied to the database of protein sequences for functional analysis can be successfully derived from the relatively small dataset of proteins and protein complexes of known structure available in the PDB. We extract the framework of contacting residues from families of similar complexes and consider the frequency of occurrence of specific residues in the identified contacting positions. We then use these data to infer interaction specificity for elements of the family for which interaction data are not yet available.

Careful analysis of structural regions surrounding residues conserved in PROSITE patterns can be used to define new patterns, extended with residues that can be identified only in a multiple alignment of true positive structures. Such new patterns, which always contain the PROSITE patterns used to select the true positives, have been shown to be able to select all the true positives, and less false positives, in a database of well-annotated protein sequences.

Moreover, we use a new method for local structure comparison to infer protein function in proteins of unknown function, or where only some of the encoded functions have already been characterized. This method is now included in a database of protein surface patches, that are annotated at the residue level for any function that we were able to use for annotation (binding for small ligands, PROSITE patterns and so on).

### References

1. Brannetti B, Helmer-Citterich M. iSPOT: a web tool to infer the interaction specificity of families of protein modules. *Nucleic Acids Res* (in press).
2. Burley SK, Bonanno JB. 2003. Structural genomics. *Methods Biochem Anal* **44**: 591–612.
3. Dower WJ, Mattheakis LC. 2002. *In vitro* selection as a powerful tool for the applied evolution of proteins and peptides. *Curr Opin Chem Biol* **3**: 390–398.
4. Falquet L, Pagni M, Bucher P, *et al.* 2002. The PROSITE database, its status in 2002. *Nucleic Acids Res* **30**: 235–238.
5. Laskowski RA. 1995. SURFNET: a program for visualizing molecular surfaces, cavities, and intermolecular interactions. *J Mol Graph* **13**(5): 323–330, 307–308.
6. Laskowski RA, Luscombe NM, Swindells MB, Thornton JM. 1996. Protein clefts in molecular recognition and function. *Protein Sci* **5**(12): 2438–2452.
7. Reimer U, Reineke U, Schneider-Mergener J. 2002. Peptide arrays: from macro to micro. *Curr Opin Biotechnol* **4**: 315–320.
8. Westbrook J, Feng Z, Chen L, Yang H, Berman HM. 2003. The Protein Data Bank and structural genomics. *Nucleic Acids Res* **31**: 489–491.

Copyright © 2003 John Wiley & Sons, Ltd. 
